# Fluorescently tagged nanobodies and NanoBRET to study ligand-binding and agonist-induced conformational changes of full-length EGFR expressed in living cells

**DOI:** 10.3389/fimmu.2022.1006718

**Published:** 2022-11-23

**Authors:** Dehan Comez, Jacqueline Glenn, Stephanie M. Anbuhl, Raimond Heukers, Martine J. Smit, Stephen J. Hill, Laura E. Kilpatrick

**Affiliations:** ^1^ Division of Physiology, Pharmacology and Neuroscience, School of Life Sciences, University of Nottingham, Nottingham, United Kingdom; ^2^ Centre of Membrane Proteins and Receptors (COMPARE), University of Birmingham and University of Nottingham, The Midlands Nottingham, Nottingham, United Kingdom; ^3^ Division of Medicinal Chemistry, Amsterdam Institute of Molecular and Life Sciences (AIMMS) Vrije Universiteit (VU), Amsterdam, Netherlands; ^4^ QVQ Holding BV, Utrecht, Netherlands; ^5^ Division of Bimolecular Science and Medicinal Chemistry, Biodiscovery Institute, School of Pharmacy, University of Nottingham, Nottingham, United Kingdom

**Keywords:** EGFR, nanobody, BRET, NanoBiT, fluorescence

## Abstract

**Introduction:**

The Epidermal Growth Factor Receptor is a member of the Erb receptor tyrosine kinase family. It binds several ligands including EGF, betacellulin (BTC) and TGF-α, controls cellular proliferation and invasion and is overexpressed in various cancer types. Nanobodies (VHHs) are the antigen binding fragments of heavy chain only camelid antibodies. In this paper we used NanoBRET to compare the binding characteristics of fluorescent EGF or two distinct fluorescently labelled EGFR directed nanobodies (Q44c and Q86c) to full length EGFR.

**Methods:**

Living HEK293T cells were stably transfected with N terminal NLuc tagged EGFR. NanoBRET saturation, displacement or kinetics experiments were then performed using fluorescently labelled EGF ligands (EGF-AF488 or EGF-AF647) or fluorescently labelled EGFR targeting nanobodies (Q44c-HL488 and Q86c-HL488).

**Results:**

These data revealed that the EGFR nanobody Q44c was able to inhibit EGF binding to full length EGFR, while Q86c was able to recognise agonist bound EGFR and act as a conformational sensor. The specific binding of fluorescent Q44c-HL488 and EGF-AF488 was inhibited by a range of EGFR ligands (EGF> BTC>TGF-α).

**Discussion:**

EGFR targeting nanobodies are powerful tools for studying the role of the EGFR in health and disease and allow real time quantification of ligand binding and distinct ligand induced conformational changes.

## Introduction

The epidermal growth factor receptor (EGFR) is a glycoprotein of 170 kDa, encoded by a gene located on chromosome 7p11.2 (1). It has a cysteine-rich extracellular region, a single transmembrane spanning region and an intracellular domain with tyrosine kinase activity (2). Its extracellular portion is subdivided into four distinct regions with domains I and III containing the sites for EGF binding and the cysteine-rich domains II and IV containing N-linked glycosylation sites. The first step of EGFR activation has been proposed to involve ligand-induced dimerization of EGFR, leading to stimulation of its intracellular kinase domain and autophosphorylation of EGFR at multiple intracellular tyrosine residues (3). This results in the recruitment of downstream signalling proteins such as Src homology domain-containing adaptor protein C (Shc), growth factor receptor-bound protein 2 (Grb2) and phospholipase Cγ (PLCγ) (1, 2, 4).

X-ray crystallography using purified extracellular regions of EGFR produced the original elegant scheme for ligand-induced EGFR dimerization (5). Binding of EGF to domains I and III stabilizes an extended conformation and exposes a dimerization interface in domain II, promoting self-association with a K_D_ in the micromolar range (5–10). However, this model does not capture the complex ligand-binding characteristics seen for cell surface full-length EGFRs in intact cells, where there is increasing evidence of negative cooperativity (7) and distinct affinity states for ligand-binding and intracellular signalling (4, 8–10).

Insight into the structural origins of EGF/EGFR binding complexity has been provided by studies of the Drosophila EGFR (dEGFR), which, unlike its human counterpart, retains its negative cooperativity when the soluble extracellular regions are isolated and purified (11). This work has shown that single ligand occupied asymmetric dimers can form (7, 11, 12). Mutations that block EGFR dimerization (Y251A and R285S) do not reduce ligand affinity (9) but do abolish EGFR signalling (6, 13). Furthermore, extracellular EGFR-activating mutations (R84K and A265V or A265D) enhance ligand-binding affinity without directly promoting EGFR dimerization, suggesting that these particular oncogenic mutations alter the allosteric linkage between dimerization and ligand binding (9).

EGFR is activated by seven different growth factors (14), which fall into two groups based on receptor-binding affinity (10). The high-affinity ligands are EGF, transforming growth factor-α (TGFα), betacellulin (BTC) and heparin binding EGF-like growth factor (HB-EGF) and the low-affinity ligands are epiregulin, epigen and amphiregulin (10). Individual EGFR ligands also induce qualitatively and quantitatively different downstream signals (15–17). Recent crystallographic and cellular studies have shown that two EGFR ligands, epiregulin and epigen, drive the purified EGFR extracellular domains into dimers, each resulting in different structures (10). The resulting ligand-induced dimers were weaker and more short-lived than those induced by EGF itself, suggesting that epiregulin and epigen are both partial agonists of EGFR dimerization (10). Unexpectedly, this weakened dimerization elicited more sustained responses than EGF, provoking responses in breast cancer cells associated with differentiation rather than proliferation (10). In addition, recent cryo-EM structures of full-length EGFR bound to EGF or TGFα have revealed differential stabilization of quaternary structures of EGFR dimers where the membrane proximal tips of domain IV are either juxtaposed or separated (18). EGF and TGFα differ in their ability to maintain the conformation with the membrane-proximal tips separated (18).

Heavy-chain antibodies have been described in species belonging to the camelid family that can target EGFR (19). Heavy chain antibodies are composed of two identical heavy chains and do not contain a light chain (19). Their antigen-binding part is therefore composed of a single immunoglobulin (Ig) variable region (VHH or nanobody) that can be easily incorporate into, and expressed from, a plasmid and genetically engineered to generate novel receptor specific probes. This approach has revealed nanobodies that bind to a similar site to EGF on the receptor (20, 21) and others that bind to EGFR but do not compete for EGF binding and are non-activating (20, 22). EgB4 is an example of the latter category of EGFR nanobody that has previously been used to evaluate gross movements of the extracellular domains of EGFR with respect to a fluorescent membrane dye (23). We have recently demonstrated for G protein-coupled receptors that receptor-specific nanobodies can be used to monitor ligand binding and conformation changes using NanoBRET technology (24). In the present study we have used N-terminal nanoluciferase-tagged EGFR and NanoBRET to investigate the pharmacological properties of a fluorescent derivative of EgB4 (Q86c-HL488) and a second fluorescent nanobody that binds to the EGF-binding site (Q44c-HL488) in a similar manner to the previously described 7D12 (21).

## Materials and methods

### Materials

Epidermal Growth Factor fluorescently labelled with Alexa Fluor 488 (E13345) or Alexa Fluor 647 (E35351) were purchased from Thermo Fischer Scientific (Waltham, USA). Human recombinant TGF-alpha (239-A-100), human recombinant betacellulin (261-CE-010), human recombinant epiregulin (1195-EP-025), human recombinant amphiregulin (262-AR-100), human recombinant epigen (6629-EP-025) and human recombinant EGF (236-EG-200) were purchased from R&D Systems (Minnesota, USA). Purified LgBiT, FuGENE HD Transfection Reagent and furimazine were purchased from Promega Corporation. Opti-MEM reduced serum medium was purchased from Gibco (31985062). Q44c and Q86c, containing an unpaired cysteine in the C-terminal tag, were provided by QVQ (Utrecht, The Netherlands).

### DNA constructs

cDNA encoding N terminal fusions of EGFR to NanoLuc or HiBiT were a kind gift from Promega Corporation, with the EGFR ORF originally obtained from the Kazusa DNA Research Institute (Kisarazu, Japan). For N-terminal NanoLuc tagged constructs, EGFR lacking its native signal sequence, was cloned into a pNKF1-secN CMV vector fusing the signal peptide sequence of IL-6 onto the N terminus of NanoLuc. The resulting vector encoded NanoLuc fused to the N-terminus of EGFR *via* a Gly-Ser-Ser-Gly (AIA) linker (termed NLuc- EGFR). For N-terminal HiBiT tagged constructs, HiBiT (VSGWRLFKKIS) was inserted after the signal peptide from IL-6 and fused to EGFR using a GSSG linker (termed HiBiT-EGFR).

### Cell culture

Human embryonic kidney (HEK293) cells stably expressing N-terminal NanoLuc-tagged EGFR (NLuc-EGFR) and wildtype HEK293 cells were cultured in Dulbecco’s Modified Eagle Medium-high glucose (DMEM; D6429, Sigma Aldrich) containing 10% fetal calf serum (FCS; F7524, Sigma Aldrich) at 37^0^C/5% CO_2_. Cells were passaged at 70% confluency using phosphate buffer saline (PBS; D8537, Sigma Aldrich) and trypsin (0.25% w/v in versene; T4174, Sigma Aldrich). All stable and transient transfections were performed using FuGENE HD (Promega Corporation) at a reagent to cDNA ratio of 3:1 following manufacturer’s instructions. We confirm that these cell lines are mycoplasma free.

### Nanobody production, purification and conjugation

Nanobodies were produced in *Saccharomyces cerevisiae* (strain *VWK18 gal1*) as described previously (25). Purification was performed using a CaptureSelect™ C-tagXL column (#494307205, Thermo Fisher Scientific) and pH elution (20 mM citric acid, 150 mM NaCl, pH=3). After dialyzing against PBS, protein purity and integrity was verified by SDS PAGE under reducing conditions, and protein concentration was determined by UV Vis measurement at 280 nm. Q44c and Q86c were site-directionally conjugated to HiLyte™ Fluor 488 C2 maleimide (AS-81164, Anaspec, Fremont, USA) using the unpaired thiol in the tag (later called Q44c-HL488 and Q86c-HL488). First, thiols were reduced using 2.75-times molar excess of Tris(2-carboxyethyl)phosphine (TCEP) (0797C437, Sigma-Aldrich) for 3 hours at 37°C. Then, a 4-times molar excess of HiLyte™ Fluor 488 C2 maleimide dissolved in DMSO was added. After 5 minutes of incubation at room temperature, the remaining TCEP and dye were removed using 2 Zeba™ desalting spin columns (89882, Thermo Fischer Scientific). Degree of labelling was determined using UV-VIS spectrometry and was >0.5. The amount of free dye was assessed upon size separation by SDS-PAGE followed by a fluorescence scan (Ex: 475 nm, Em ≥ 520 nm, D-Digit Scanner, LI-COR Biosciences, Lincoln, USA) and was<5%.

### NanoBRET ligand and nanobody saturation binding assays

HEK293 cells stably expressing NLuc-EGFR were seeded onto poly-D-lysine coated (Sigma Aldrich; 0.1 mg·mL^−1^) 96-well flat bottom, μCLEAR® white CELLSTAR® TC plates (Greiner Bio-One 655098, Stonehouse, UK) in 100 µL DMEM, at a density of 40,000 cells/well. Plates were incubated at 37°C /5% CO_2_ overnight. The next day, culture media was removed, and each well washed with 100 µL of HEPES buffered Salt Solution (HBSS) (2 mM of sodium pyruvate, 146 mM of NaCl, 5 mM of KCl, 1 mM of MgSO_4_.7H_2_O, 10 mM of HEPES, 1.3 mM of CaCl_2_.2H_2_O, 1.5 mM NaHCO_3_, 10 mM D-glucose; pH 7.45) containing 0.2% BSA. After this washing step, fluorescently labelled EGF ligands (0-100nM) or nanobodies (0-200nM) were added to the appropriate wells in increasing concentrations (in the presence or absence of 100nM EGF) in 50 µL total volume of HBSS per well. Cells were incubated in the dark at 37°C for 30 minutes. 12.5 nM final concentration of furimazine was added to each well and cells were incubated for a further 5 minutes. Fluorescence and luminescence emissions were simultaneously detected using a PHERAstar FS dual plate reader (BMG Labtech, Offenburg, Germany). When using red fluorescently labelled EGF (EGF-AF647) emissions were detected using an optic module fitted with a 460 nm (80 nm) bandpass filter for collecting luminescence (NLuc) emissions and a >610 nm long pass filter for fluorescence emissions (AF647). For green fluorescently labelled EGF (AF488) or labelled nanobodies (Q44-HL488 or Q86-HL488) emissions were detected using an optic module fitted with a 475 nm (30 nm) band-pass filter for collecting luminescence emissions and a 535 nm (30 nm) band pass filter for fluorescence emissions (AF488). Raw BRET ratios were calculated by dividing fluorescence emissions by luminescence emissions, and the results were plotted using GraphPad Prism 9.2 (GraphPad Software, La Jolla, CA).

### NanoBRET nanobody displacement assay

HEK293 cells stably expressing NLuc-EGFR (40,000/well) were plated onto poly-D-lysine-coated white 96-well plates as described above. After overnight incubation at 37^0^C/5% CO_2,_ cells were washed with HBSS containing 0.2% BSA. Increasing concentrations of non-fluorescent ligands or nanobodies were simultaneously added alongside a fixed concentration of fluorescent EGF (EGF-AF488 or EGF-AF647) or nanobody (HL488 tagged) to each well in a 50 µL final volume of HBSS containing 0.2% BSA. Cells were incubated for 30 minutes at 37°C/5% CO_2_ in the dark. A 12.5 nM final concentration of furimazine was added to each well. Fluorescence and luminescence were measured simultaneously using a PHERAstar FS dual plate reader as described previously.

### Nanobody kinetics assay

HEK293 cells stably expressing NLuc-EGFR (40.000/well) were plated onto poly-D-lysine-coated white 96-well plates as described above and incubated overnight at 37^0^C/5% CO_2_. The next day cells were washed with 100 µL of HBSS containing 0.2% BSA. 45 µL of HBSS containing furimazine (12.5 nM final concentration) was added to each well. Baseline BRET measurements were undertaken using a PHERAstar FS dual plate reader for 15 minutes at 37°C every 60 seconds. After baseline measurement, fluorescent nanobodies (3.125 – 200nM) were added to the cells. Plates were read for 2 hours, every 60 seconds at 37°C. For EGF competition assays, increasing concentrations of non-fluorescent EGF (10^-13^ – 10^-7^M) were added 30 minutes after nanobody addition and measurements continued for a further 90 minutes at 37°C using a BRET 1 plus optical module.

### NanoBiT internalization assay

HEK293 cells (20,000/well) were plated onto poly-D-lysine-coated white 96-well plates as described previously and incubated at 37°C/5% CO_2_ overnight. The next day cells were transfected with 100 ng per well of HiBiT-EGFR cDNA with FuGENE HD Transfection Reagent using a 3:1 DNA/FuGENE HD ratio in OptiMEM following manufacturer’s instructions. Cells were then incubated at 37°C/5% CO_2_ overnight. The next day culture media was removed, and cells were washed with HBSS once. Cells were incubated with 100nM EGF, Q44 or Q86 nanobodies in HBSS containing 0.02% BSA for 120, 60, 30 or 5 minutes at 37°C. Plates were then washed once using HBSS/0.02% BSA and then incubated with 10 nM of purified LgBiT and furimazine (1/400 dilution) diluted in HBSS/0.02% BSA for 20 minutes. Luminescence was then measured using a PHERAstar FS plate reader (BMG Labtech, Offenburg, Germany) using the LUM Plus optical module.

### Data analysis

All data obtained from NanoBRET assays were determined from BRET ratios calculated using Microsoft Excel:



$$\text{B}RET\;\text{ratio}=\frac{\text{Emission from acceptor channel}}{\text{Emission from donor channel}}$$

Data were analysed using GraphPad Prism 9.20 (GraphPad Software, La Jolla, CA, USA). Data are presented as mean ± S.E.M. All experiments were performed in 5-6 independent experiments with triplicate wells (see figure legends for details). Drug additions were randomly allocated to wells within each 96-well plate. Statistical significance was defined as *P<*0.05.

Saturation binding curves were fit to the following equation:


$$Total\;Binding=\;B_{MAX}.\frac{\;\left[L\right]}{\left[L\right]+\;K_{D}}+M.\left[L\right]+C$$

where [L] is the concentration of fluorescent ligand (nanobody or EGF), B_MAX_ is the level of maximal specific binding, K_D_ is the equilibrium dissociation constant of the labelled ligand in the same units as [L], M is the slope of the non-specific binding component, C represents the background BRET ratio (in the absence of fluorescent ligand). In the case of EGF-AF488 or EGF-AF647, total and non-specific binding (obtained in the presence of 100 nM EGF) were fitted simultaneously with shared parameters for M and C. In the case of Q86c-HL488, total binding curves obtained in the presence or absence of 100 nM EGF were fitted simultaneously to the above equation with shared parameters for M and C.

Competition binding data were fit to following equation:


$$\%\;Inhibition\;of\;specific\;binding=\frac{\left(100\times \left[A\right]\right)}{\left(\left[A\right]\times IC_{50}\right)}$$

where [A] is the concentration of unlabelled ligand and *IC_50_* is the concentration of ligand required to inhibit 50% of the specific binding of the fluorescent ligand. In the case of EGF-AF488 and EGF-AF647 competition experiments, the *IC_50_* values were then used to calculate the K_i_ values using the Cheng-Prussoff equation:


$$K_{i}=\frac{IC_{50}}{1+\frac{\left[L\right]}{K_{D}}}$$

where [L] is the concentration of fluorescent ligand in nM, and *K_D_* is the dissociation constant of that fluorescent ligand in nM.

In the case of Q86c-HL488 binding experiments where increasing concentrations of EGFR ligands produced a marked increase in the level of specific binding, the data were fit to the following equation:


$$\%\;Increase\;in\;specific\;binding=\frac{\left(100\times \left[A\right]\right)}{\left(\left[A\right]\times EC_{50}\right)}$$

where [A] is the concentration of unlabelled EGFR ligand and *EC_50_* is the concentration of ligand required to produce 50% of the maximum increase in the specific binding of the fluorescent ligand.

For HiBiT internalization experiments, all data were normalised to relative luminescence units obtained for buffer only (HBSS/0.02% BSA; 100%) for each individual experiment. Normalised data across experimental replicates were then pooled and statistical significance was determined using one way ANOVA and defined as *P<*0.05.

## Results

### Effect of Q44c and Q86c on EGF ligand binding to NLuc-EGFR

**Figure 1:**
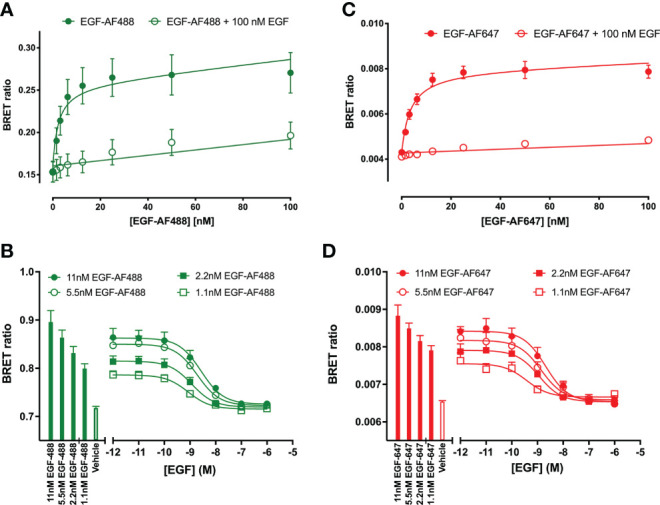
Quantification of fluorescent EGF binding to HEK293 cells stably expressing a full-length N-terminal nanoluficerase (NanoLuc) tagged-EGFR measured using NanoBRET. Saturation binding of fluorescently labelled **(A)** EGF-AF488 and **(C)** EGF-AF647 in the absence (closed circles) or presence (open circles) of 100 nM unlabelled EGF added simultaneously and incubated for 30 minutes at 37^o^C. Saturation experiments were performed in HBSS containing 0.2 % BSA. Displacement of various fixed concentrations of **(B)** EGF-AF488 or **(D)** EGF-AF647 by increasing concentrations of unlabelled EGF. Both ligands were added simultaneously, and cells incubated for 60 minutes at 37^o^C. The NLuc substrate furimazine (12.5 nM) was added and plates incubated for 5 minutes then luminescence and fluorescence emissions were measured using a BMG Pherastar. Displacement experiments were performed in HBSS containing 0.1 % BSA. Closed bars represent fluorescent EGF alone, with open bars representing vehicle (HBSS/0.1% BSA). Data are combined mean ± SEM from five independent experiments, where each experiment was performed in triplicate.

**Figure 2:**
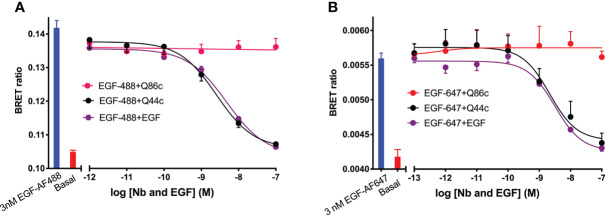
Displacement of **(A)** EGF-AF488 (3 nM) or **(B)** EGF-AF647 (3 nM) by unlabelled-EGFR nanobodies Q44c, Q86c and unlabelled EGF. NanoBRET experiments were performed using HEK293 cells stably expressing full-length N-terminal nanoluciferase-EGFR. Fluorescently labelled EGF and competing unlabelled ligands were added simultaneously and incubated for 30 minutes at 37^o^C. Experiments were performed in HBSS containing 0.2% BSA. The NLuc substrate furimazine (12.5 nM) was added and plates incubated for 5 minutes then luminescence and fluorescence emissions were measured using a BMG Pherastar. Blue bars represent BRET ratios obtained for total EGF-AF488 or EGF-AF647 binding in the absence of competing ligand, whereas red bars represent those measured for HBSS/0.2% BSA buffer alone (basal). Data are combined mean ± SEM from five independent experiments, where each experiment was performed in triplicate.

**Figure 3:**
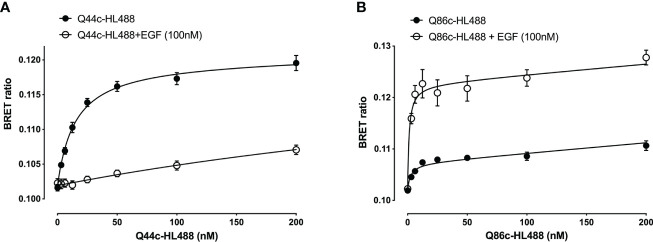
Saturation binding of fluorescently (HiLyte^TM^ Fluor 488) labelled EGFR nanobodies **(A)** Q44c-HL488 and **(B)** Q86c-HL488 in absence (closed circles) or presence (open circles) of 100 nM EGF. NanoBRET experiments were performed using full-length N-terminal nanoluciferase-EGFR stably expressing HEK293 cells. Nanobodies and EGF were added simultaneously and incubated for 30 minutes at 37^o^C. Experiments were performed in HBSS containing 0.2 % BSA. The NLuc substrate furimazine (12.5 nM) was added and plates incubated for 5 minutes then luminescence and fluorescence emissions were measured using a BMG Pherastar. Data are combined mean ± SEM from five independent experiments, where each experiment was performed in triplicate.

Initial studies were undertaken to investigate the effect of unlabelled Q44c and Q86c on the binding of fluorescent analogues of EGF to the full-length EGFR receptor expressed in living HEK293 cells. Both EGF-AF488 and EGF-AF647 exhibited saturable binding to the N-terminal nanoluciferase-tagged EGFR (NLuc-EGFR) that was displaceable by 100 nM unlabelled EGF (**Figures 1A, C**). The mean K_D_ values obtained for EGF-AF488 and EGF-AF647 were 2.30 ± 0.09 nM (n=5) and 3.49 ± 0.21 nM (n=5) respectively. Furthermore, increasing concentrations of unlabelled EGF were able to potently inhibit the specific binding of different concentrations of EGF-AF488 (**Figure 1B**) and EGF-AF647 (**Figure 1D**) yielding pKi values for unlabelled EGF of 9.35 ± 0.02 (n=5) and 9.61 ± 0.06 (n=5) respectively. Consistent with Q44c binding to the same epitope as EGF on the EGFR, this nanobody was able to potently displace the binding of both 3 nM EGF-AF488 (pIC_50_ = 8.63 ± 0.05, n=5; **Figure 2A**) and 3 nM EGF-AF647 (pIC_50_ = 8.61 ± 0.15, n=5; **Figure 2B**). In contrast, Q86c showed no significant effect on the binding of both fluorescent EGF analogues at concentrations up to 100 nM (**Figures 2A, B**).

### Binding of fluorescent nanobodies Q44c-HL488 and Q86c-HL488 to NLuc-EGFR

Next, the ability of fluorescently labelled Q44c and Q86c to bind to full-length N-terminal nanoluciferase-EGFR was investigated using NanoBRET. Genetic introduction of a C-terminal cysteine residue into the nanobody sequence allowed a directional attachment of a fluorophore (HiLyte Fluor488; HL488) to both nanobodies without affecting their binding properties. Both Q44c-HL488 and Q86c-HL488 were able to bind to NLuc-EGFR and demonstrated a clear saturability of specific binding. Analysis of the ligand-binding isotherms assuming that there was both a saturable component of specific binding and a linear component of non-specific binding revealed K_D_ values of 14.94 ± 1.04 nM (n=5) and 3.21 ± 1.10 nM (n=5) for Q44c-HL488 and Q86c-HL 488 respectively (**Figure 3**). In the presence of 100 nM EGF, the specific binding of Q44c-HL488 was completely prevented leaving only the expected linear non-specific component of binding (**Figure 3A**). In marked contrast, in the presence of 100 nM EGF the specific binding of Q86c-HL488 was markedly enhanced leading to a significant increase (437.6 ± 57.3%, n=5) in the B_MAX_ value (p<0.005; paired t test) compared to that obtained in the absence of EGF (**Figure 3B**). In addition, the K_D_ value of Q86c-HL488 obtained in the presence of 100 nM EGF was slightly decreased (1.18 ± 0.28 nM, n=5).

**Figure 4:**
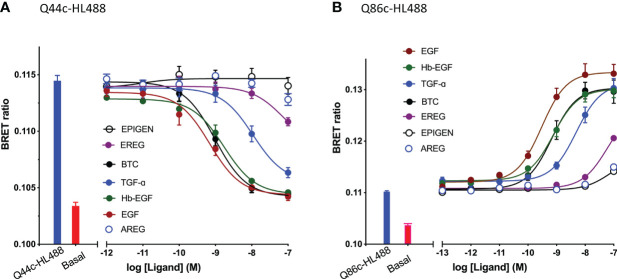
Effect of EGFR ligands on the binding of fluorescent **(A)** Q44c-HL488 (14.6 nM) and **(B)** Q86c-HL488 (12.5 nM) to full-length N-terminal nanoluciferase-EGFR stably expressed in HEK293 cells. Cells were treated with nanobodies and EGFR ligands simultaneously and incubated for 30 minutes at 37^o^C. Experiments were performed in HBSS containing 0.2 % BSA. The NLuc substrate furimazine (12.5 nM) was added and plates incubated for 5 minutes then luminescence and fluorescence emissions were measured using a BMG Pherastar. Blue bars represent BRET ratios obtained for total Q44c-HL488 or Q86c-HL488 in the absence of competing ligand, whereas red bars represent those measured for HBSS/0.2% BSA buffer alone (basal). Data are combined mean ± SEM from five independent experiments, where each experiment was performed in triplicate.

The specific binding of Q44c-HL488 was inhibited by a range of EGF ligands with a rank order of potency of EGF>BTC=Hb-EGF>TGF-α>ERG>AREG and Epigen (**Figure 4A** and **Table 1**). A very similar rank order of potency was obtained with these ligands for their enhancement of the specific binding of Q86c-HL488 to NLuc-EGFR (**Figure 4B** and **Table 1**). Thus, molecules that bind with higher affinity to the EGF binding site are more efficient in modifying the binding of Q86 to EGFR. The rank order of potencies was also comparable to that obtained from inhibition of the binding of 3 nM EGF-AF488 (**Table 1**), although the actual EC_50_ and IC_50_ values for modulating the binding of both Q44c-HL488 and Q86c-HL488 were at lower concentrations than the pKi value calculated from displacement of EGF-AF488 binding (**Table 1**). This was most marked for TGF-α and probably confirms that the EC_50_ and IC_50_ values also relate to agonist efficacy and the consequences of receptor activation and conformational changes.

**Table 1:** pIC_50_ and pEC_50_ Values for the effect of EGFR ligands on the binding of 14.6 nM Q44c-HL488 or 12.5 nM Q86c-HL488 to full-length N-terminal nanoluciferase-EGFR in HEK293 Cells.

EGFR Ligand	Q44c-HL488 (pIC_50_)	Q86c-HL488 (pEC_50_)	EGF-AF488 (pKi)
EGF	9.23 ± 0.11 (n=5)	9.52 ± 0.06 (n=5)	8.86 ± 0.07 (n=5)
Hb-EGF	8.80 ± 0.13 (n=5)	9.20 ± 0.17 (n=5)	8.43 ± 0.08 (n=5)
TGF-α	7.96 ± 0.19 (n=5)	8.32 ± 0.09 (n=5)	6.83 ± 0.05 (n=5)
BTC	9.02 ± 0.14 (n=5)	9.17 ± 0.09 (n=5)	8.45 ± 0.05 (n=5)

Values are mean ± S.E.M of n individual experiments. These values have also been compared with their pKi values determined from inhibition of binding of 3 nM EGF-AF488.

**Figure 5:**
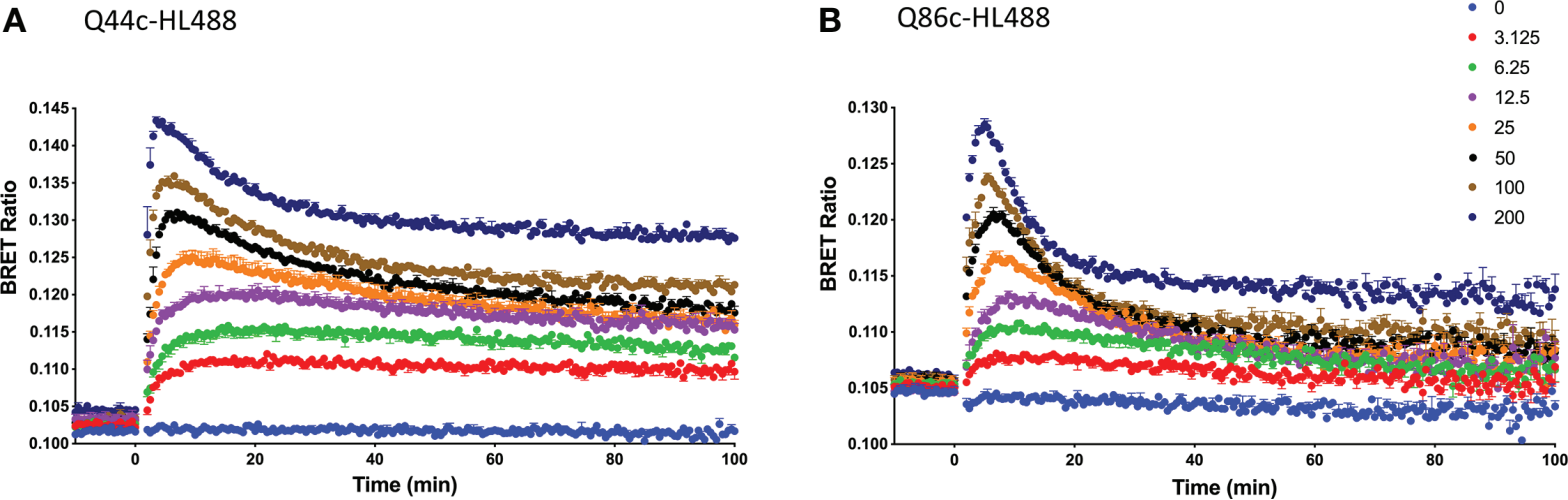
Kinetic NanoBRET experiment showing the binding of different concentrations of fluorescent **(A)** Q44c-HL488 or **(B)** Q86c-HL488 to full-length N-terminal nanoluciferase-EGFR. The concentrations of Q44c-HL488 and Q86c-HL488 are given in nM. HEK293 cells stably expressing NLuc-EGFR were treated with furimazine (12.5 nM) and luminescence and fluorescence values were read for 15 minutes (every 60 sec) at 37^o^C using a BMG Pherastar. Experiments were performed in HBSS containing 0.2 % BSA. Following this period, cells were treated with various concentrations of either fluorescent nanobody and the luminescence and fluorescence emissions simultaneously recorded for a further 100 min at 37^o^C. Data are mean ± SEM from triplicate determinations in a single experiment. This single experiment is representative of five independent experiments performed.

**Figure 6:**
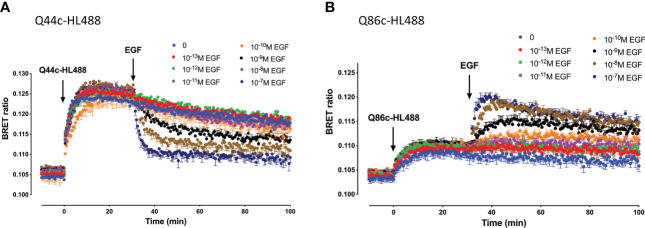
Kinetics of EGF-induced changes in the NanoBRET signal obtained with fluorescent **(A)** Q44c-HL488 or **(B)** Q86c-HL488 binding to NLuc-EGFR. HEK293 cells stably expressing full-length N-terminal nanoluciferase-EGFR were treated with furimazine (12.5 nM) and, luminescence and fluorescence values were read for 15 minutes (every 60 sec) at 37^o^C using a BMG Pherastar. Following this period, cells were treated with 25 nM of either respective fluorescent nanobody and luminescence and fluorescence emissions simultaneously recorded for a further 30 min at 37^o^C. After 30 minutes, various concentrations of EGF were added to the wells and measurements continued for a further 30 minutes at 37^o^C. Data are mean ± SEM from triplicate determinations in a single experiment. This single experiment is representative of five independent experiments performed.

Kinetic analysis of the binding of both Q44c-HL488 and Q86c-HL488 to NLuc-EGFR indicated that at the higher concentrations of fluorescent nanobody used in these experiments, two components were observed in their kinetic profiles represented by a fast pronounced peak in the BRET ratio followed by a decline to a lower plateau (**Figures 5A, B**). This was more marked for Q86c-HL488 (**Figure 5B**) and might suggest a time-dependent change in receptor conformation or the onset of a component of receptor internalisation. Addition of EGF after a steady plateau of binding had been achieved with 25 nM fluorescent nanobody, yielded an expected inhibition (**Figure 6A**) or stimulation (**Figure 6B**) of Q44c-HL488 and Q86c-HL488 binding to EGFR respectively. Interestingly, the stimulatory effect of EGF on Q86c-HL488 was characterised by a slow fall to a lower plateau after the initial peak was obtained.

### Interactions between Q44c and Q86c

**Figure 7:**
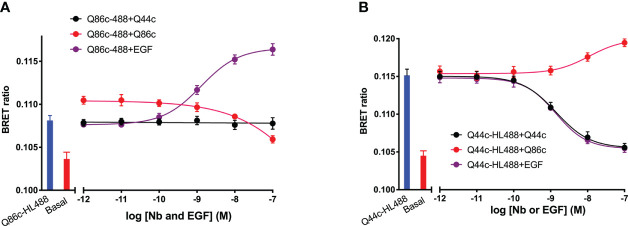
The effect of unlabelled Q44c, Q86c and EGF on the binding of fluorescent **(A)** Q44c-HL488 (14.6 nM) and **(B)** Q86c-HL488 (12.5 nM) to full-length N-terminal nanoluciferase-EGFR. NanoBRET experiments were performed using NLuc-EGFR stably expressing HEK293 cells. Cells were treated with either nanobody and EGF simultaneously and incubated for 30 minutes at 37^o^C. The NLuc substrate furimazine (12.5 nM) was added and plates incubated for 5 minutes then luminescence and fluorescence emissions were measured using a BMG Pherastar. Experiments were performed in HBSS containing 0.2 % BSA. Data are combined mean ± SEM from five independent experiments, where each experiment was performed in triplicate.

To evaluate whether there were interactions between Q44c and Q86c in binding to full-length N-terminal nanoluciferase-EGFR we undertook competition binding experiments with their fluorescent analogues. Unlabelled Q44c had no effect on the specific binding of Q86c-HL488 under conditions where the positive effect of EGF could be clearly demonstrated (**Figure 7A**). Q86c did, however, produce an inhibition of Q86c-HL488 binding at the highest concentrations used. As expected, both unlabelled Q44c and EGF inhibited the binding of Q44c-HL488 consistent with the proposal that Q44c and EGF bind to the same epitope of EGFR (**Figure 7B**). Interestingly, Q86c was able to produce a small but significant (p<0.05; One-way ANOVA) enhancement of Q44c-HL488 binding (**Figure 7B**) reminiscent of the effect of the low affinity EGF ligands on the binding of Q86c-HL488 (**Figure 4B**).

### Effect of unlabelled Q44c and Q86c on EGFR internalization measured using NanoBiT

**Figure 8:**
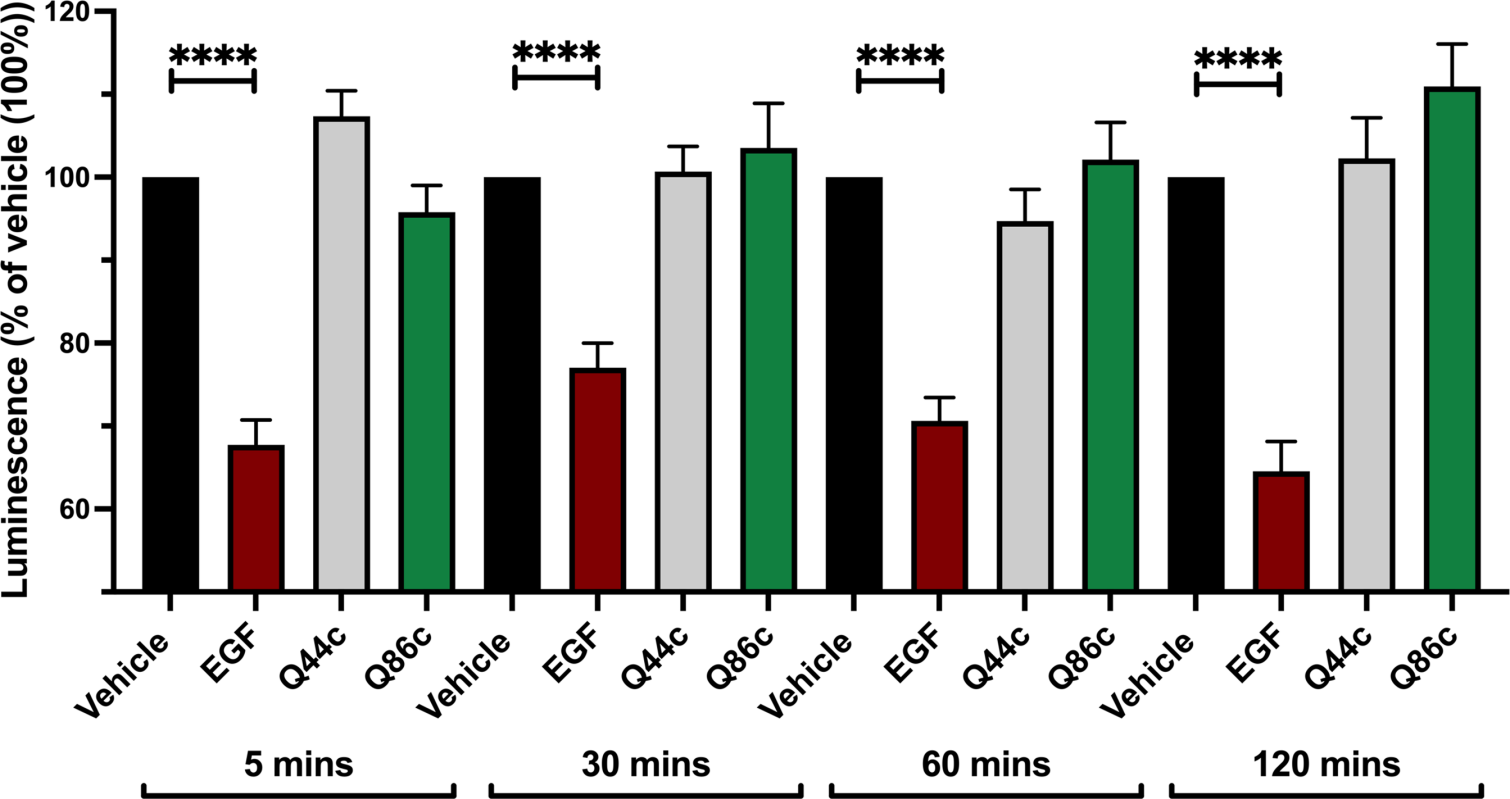
Effect of unlabelled Q44c, Q86c and EGF on EGFR internalization measured using NanoBiT. HEK293 cells transiently expressing HiBiT-EGFR cDNA, were treated with EGF (100 nM), Q44c (100 nM) or Q86c (100 nM) in HBSS/0.02% BSA for 5, 30, 60 or 120 minutes at 37^o^C. Purified LgBiT (10 nM) and furimazine (1:400 dilution) were then added and cells incubated for a further 20 minutes at 37^o^C to allow NanoBiT recomplementation (leading to the formation of full-length nanolucifierase) and furimazine oxidation to occur. Luminescence emissions were then measured using a BMG Pherastar. Data are mean ± SEM from quadruplicate observations in a single experiment pooled from 5 (120 minutes incubation) and 7 (5, 30, 60 minute incubations) independent experiments. Data were normalized to vehicle controls (100%) and statistical significance determined using a one way ANOVA (**** = P<0.0001).

To determine whether the fall to a plateau from an initial peak in the kinetic profile of Q44c-HL488 and Q86c-HL488 binding was due to the onset of receptor internalization, we studied EGFR internalization in response to unlabelled EGF, Q44c or Q86c using N-terminal HiBiT-tagged EGFR (26). In this approach, purified LgBiT is added after the agonist stimulation period. As LgBiT is itself not membrane permeable, luminescence detected from re-complemented full length nanoluciferase is indicative of the EGFR population still remaining at the cell surface after agonist stimulation. Using this approach, 100 nM EGF induced significant receptor internalization within 5 min of agonist administration which was not seen with Q86c and Q44c (**Figure 8**). These data suggest that the fall in luminescence at high concentrations of both Q44c-HL488 and Q86c-HL488 following attainment of the initial peak is more likely a consequence of molecular rearrangement. However, the fall in signal from Q86c-HL488 following EGF addition could be explained by EGF-induced receptor internalization.

## Discussion

In the present study we have used small (*circa* 15 KDa) fluorescent camelid nanobodies targeting the human EGFR to investigate ligand-binding and conformational changes induced by agonists in living cells. Q44c-HL488 binds to ligand-free EGFR but not to EGF-occupied receptors suggesting that it binds to an epitope close to the EGF-binding site on domain III and can sterically interfere with the binding of EGF (20, 21). To test this directly, we used a N-terminal nanoluciferase-tagged variant of EGFR (NLuc-EGFR) expressed in living cells and monitored the binding of fluorescent variants of EGF and Q44c using NanoBRET (27–29). The proximity requirements (<10 nm) of the NanoBRET approach provided a very sensitive measure of specific binding to EGFR. Q44c-HL488 was able to directly bind to ligand-free EGFR with high affinity, and unlabelled Q44c was able to compete for specific EGF-AF488 binding to EGFR. The specific binding of Q44c-HL488 to EGFR was fully displaced by 100 nM unlabelled EGF and 100 nM non-fluorescent Q44c was able to fully prevent the binding of either EGF-AF488 or EGF-AF647 to NLuc-EGFR. In keeping with previous observations (10), other higher-affinity EGFR ligands (TGFα, BTC, HB-EGF) potently inhibited the binding of Q44c-HL488 whilst low-affinity ligands epiregulin, epigen and amphiregulin were considerably weaker with only epiregulin producing a small but significant displacement of Q44c-HL488 binding when used at a maximal concentration (100nM). Q44c-HL488 binding could, however, be completely prevented by unlabelled Q44c.

In marked contrast to the data obtained with Q44c-HL488, Q86c-HL488 was able to bind with high affinity to both ligand-free and EGF-occupied receptors. Also, unlabelled Q86c had no effect on the binding of EGF-AF488 or EGF-AF647 to NLuc-EGFR at concentrations up to 100nM. Similarly, EGF did not inhibit the binding of Q86c-HL488 to EGFR. EGF did, however, produce a marked enhancement (438%) of the BRET signal obtained with Q86c-HL488. This was mimicked in a concentration-dependent manner by all EGFR ligands with EGF, HB-EGF, BTC and TGF-α being the most potent, epiregulin producing a modest response and both epigen and amphiregulin producing very weak but observable stimulations at the highest concentrations employed (100 nM). The most likely explanation for this significant increase in Q86c-HL488 BRET induced by EGFR ligands is that it represents a conformational change related to the agonist-induced extended conformation of EGFR and exposure of the dimerization interface in domain II leading to receptor homodimerization (5–10). This is in keeping with the recent receptor X-ray crystal structures of Q86 (EgB4) alone and bound to the full extracellular EGFR-EGF complex in its extended active conformation (30)). It is also interesting that Q86c can induce a small enhancement of Q44c-HL488 binding (but not EGF-AF488 or EGF-AF647 binding) which suggests that there are subtle differences in the binding of EGF and Q44c to domain III of EGFR.

NanoBRET is dependent upon both close proximity (<10 nm) but also the orientation of the donor and acceptor moieties. Thus, conformation changes can have a profound impact on both the relative orientation and proximity of the donor and acceptor elements of the proteins of interest (29, 31). Furthermore, if as expected EGFR dimerization is induced (32), then there is also scope for additional BRET between the Q86c-HL488 and the N-terminal nanoluciferase on the opposing as well as the same protomer, resulting in an enhancement of the final BRET signal observed. The differences in the final BRET signal observed with high affinity and low affinity EGFR ligands might therefore reflect differences in the structure of the dimers generated (10) in addition to the affinity differences observed for binding to EGFR. Q86 (EgB4) has been shown previously to not compete for EGF-binding to EGFR (20, 22). Furthermore, in keeping with its ability to sense conformational changes in EGFR reported here, Q86 (EgB4) has previously been used to evaluate gross movements of the extracellular domains of EGFR from the plane of the cell membrane (23). This would be consistent with the detection of an extended conformation capable of forming homodimers.

The kinetic analysis of the binding of both Q44c-HL488 and Q86c-HL488 to NLuc-EGFR indicated that at the higher concentrations of nanobody used in these experiments, two components were observed in their kinetic profiles represented by a fast pronounced peak in the BRET ratio followed by a decline to a lower plateau. This would be consistent with either some limited conformation rearrangement of EGFR following binding of the nanobody or a nanobody-induced receptor internalisation. Furthermore, addition of EGF after a steady plateau of binding had been achieved yielded the expected inhibition (for Q44c-HL488 binding) or stimulation (for Q86c-HL488 binding). The two phases were most apparent for Q86c-HL488 and it is interesting that the subsequent stimulatory effect of EGF was characterised by a fall to a lower plateau after an initial rapid peak was obtained. If a conformational rearrangement is responsible for this effect then it is likely that this is a consequence of negative cooperativity (4, 7–10) across the dimer interface. For example, an overshoot of ligand binding to an extended active EGFR conformation could occur before the asymmetric dimers are formed leading to loss of nanobody or EGF from one of the protomers due to negative cooperativity across the dimer interface. Similarly, there is evidence for ligand-independent dimerization of non-active EGFRs which is dependent upon close proximity of the intracellular juxtamembrane domains (33–35). Negative cooperativity across the juxtamembrane dimer interface of non-active EGFRs could also explain the complex kinetic profiles of the binding of Q44c-HL488 and Q86c-HL488 in the absence of EGF.

The simplest explanation, however, for the fast pronounced peak in the BRET ratio followed by a decline to a lower plateau observed with EGF, Q44c-HL488 and Q86c-HL488 is that they are inducing a rapid internalization of a proportion of the cell surface receptors. In order to investigate this, we took advantage of the NanoBiT internalization assay developed by Soave et al (26). In this approach a small eleven amino acid fragment of nanoluciferase (HiBiT) (26, 36, 37) was added to the N-terminus of EGFR. Following stimulation of EGFR with EGF or nanobody, the large 156 amino acid fragment (LgBiT) of nanoluciferase was added to allow complementation of full length nanoluciferase and reinstatement of the luminescence signal following addition of furimazine (26, 35). Since LgBiT is cell impermeable, luminescence provides a good measure of the population of EGFR remaining at the cell surface (26, 37). This approach was able to confirm that EGF can induce internalization of the EGFR receptor. The effect of EGF was rapid (occurring within 5 min) and sustained over the 120 min of the experiment. These data are in keeping with the well-established internalization of EGFR after agonist treatment (38–40). However, internalization was not seen with the two nanobodies Q86c and Q44c. These data suggest that the fall in luminescence at high concentrations of both Q44c-HL488 and Q86c-HL488 following attainment of the initial peak is more likely a consequence of conformational change and allosteric regulation which might alter the stability of the nanobody at its binding site. However, the fall in signal from Q86c-HL488 following EGF addition could be secondary to EGF-induced receptor internalization.

**Figure 9:**
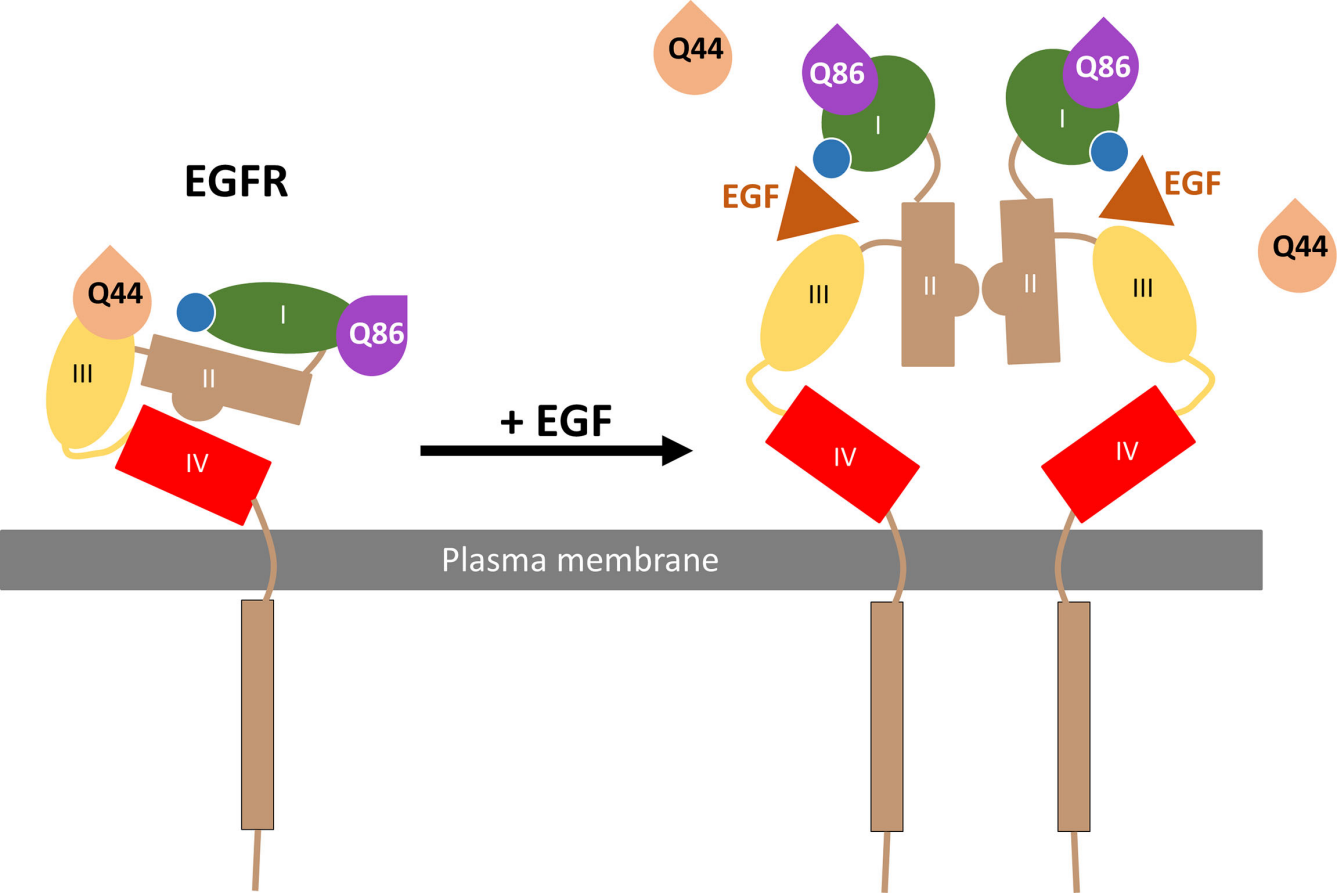
Schematic of Q44c and Q86c binding on domain III and I of the EGFR respectively. While EGF binding to the EGFR alters the EGFR conformation and causes the dissociation of Q44c, this conformational rearrangement leads to closer proximity of Q86c with the nanoluciferase tag (illustrated by the blue circle) on EGFR.

In summary, the present manuscript has used NanoBRET and NanoBiT technologies in combination with fluorescent nanobodies to demonstrate the direct binding of Q44c-HL488 and Q86c-HL488 to two different sites on the full-length EGFR receptor in living cells (**Figure 9**). Q44c-HL488 was able to inhibit EGF binding to full length EGFR consistent with the proposal that it binds to domains I and III of EGFR in a similar manner to 7D12 (21) (**Figure 9**). In contrast, Q86c-HL488 can bind to EGF-bound EGFR and act as a conformational sensor by detecting the change to an open conformation of the receptor on EGF binding (**Figure 9**). This conformational change reveals the dimerization domain II and facilitates EGFR dimerization. These data suggest that these two nanobodies will be powerful tools for studying the role of EGFR in both health and disease.

## Data availability statement

The original contributions presented in the study are included in the article/supplementary material. Further inquiries can be directed to the corresponding authors.

## Author contributions

Conceived the study: SH and LK. Generated reagents: DC, JG, SA, LK, and MS. Participated in research design: DC, SH, LK, JG, RH, SA, and MS. Conducted experiments: DC, JG, and LK. Performed data analysis: DC, SH, and LK. Wrote or contributed to the writing of the manuscript: DC, SH, LK, RH, SMA, and MJS. All authors contributed to the article and approved the submitted version.

## Funding

Research was supported by MRC (grant numbers MR/N020081/1 and MR/W016176/1) and the ONCORNET 2.0 (ONCOgenic Receptor Network of Excellence and Training 2.0) PhD training programme (DC and SA) funded by the European Commission for a Marie Sklodowska Curie Actions (H2020-MSCA grant agreement 860229). LK is funded by a University of Nottingham Anne McLaren Research Fellowship.

## Acknowledgments

The authors would like to thank Promega Corporation for kindly providing the NanoLuc-EGFR and HiBiT-EGFR constructs.

## Conflict of interest

RH is CSO of QVQ Holding B.V. and SA is affiliated to QVQ Holdings.

The remaining authors declare that the research was conducted in the absence of any commercial or financial relationships that could be construed as a potential conflict of interest.

## Publisher’s note

All claims expressed in this article are solely those of the authors and do not necessarily represent those of their affiliated organizations, or those of the publisher, the editors and the reviewers. Any product that may be evaluated in this article, or claim that may be made by its manufacturer, is not guaranteed or endorsed by the publisher.
